# Supplementary material for: Are carbon emissions trading and green financial instruments synergistic? -Comprehensive quantitative research based on content analysis
Source: PLoS One. 2024 Mar 7;19(3):e0298601. doi: 10.1371/journal.pone.0298601 (PMC10919601; doi:10.1371/journal.pone.0298601)
Supplement: S1 Data — (ZIP) [file pone.0298601.s001.zip › Data/地级市Z_F工作报告.zip!/┴╔─■/╜⌡╓▌╩╨/└┤╫╘╣1⁄2╓┌║┼í╛┬φ┐╦╩2╛▌═°í┐_2008.docx]

各位代表：

　　现在，我代表市政府向大会报告工作，请予审议，并请政协委员提出意见。

　　一、过去五年工作回顾和2007年工作情况

　　2003年以来，全市人民在省委、省政府和市委的正确领导下，坚持以邓小平理论和“三个代表”重要思想为指导，全面落实科学发展观，深入实施工业立市、以港兴市、开放牵动三大战略，开拓创新，奋力拼搏，全市经济社会保持了平稳快速发展的良好态势。特别是市委九届八次全会明确提出“三年大变样、十年大振兴”奋斗目标以来，全市上下紧紧围绕“百姓腰包鼓起来、保障体系建起来、城乡环境美起来、社会治安好起来和文化氛围浓起来”的具体目标，全力实施“工业倍增、农业升级、城市南扩、建设商贸中心、打造文化名城、构建和谐锦州”六大举措，聚精会神搞建设，一心一意谋发展，辽西沿海经济区中心城市建设取得突破性进展，较好完成了本届政府确定的各项目标和任务。

　　——国民经济持续快速增长。初步核算，地区生产总值年均增长15.4%，总量是2002年的2.4倍，超过预期121.9亿元；人均生产总值达到17845元；全社会固定资产投资累计完成530.5亿元，是上一个五年的2.3倍；地方财政一般预算收入年均增长15.8%，比2002年增加13.5亿元。三次产业结构从27.8∶29.9∶42.3调整为20.5∶39.9∶39.6。农业结构、增长方式、经营模式全面升级。工业经济高速增长，规模以上工业总产值翻两番，年均增长30.7%，超过预期18.7个百分点；增加值年均增长28.9%，超过预期16.9个百分点。区域性商贸中心优势日益显现，社会消费品零售总额年均增长11.2%。民营经济快速壮大，占地区生产总值比重达到73.3%。

　　——改革开放取得重大突破。国企改革顺利推进，314户企业完成改制任务，铁合金、金纸、华光、新华石英等企业改制后焕发出勃勃生机。农村土地延包政策得到全面落实。事业单位改革稳步实施，文化体制改革有序推进，工资改革、社会保障制度改革试点等顺利完成。累计引进外资项目263个，实际利用外资4.14亿美元，年均增长29.7%；出口创汇26亿美元，年均增长56.9%。投资环境不断优化，荣获“中国投资环境百佳城市”和“中国最具投资价值新锐金融生态城市”等称号。

　　——城乡环境建设日新月异。城市南扩步伐加快，渤海大道、南山绿化、市府广场、实验学校、第三医院、女儿河带状公园等十大南扩工程相继完成，城市骨架逐渐拉开。新建、拓宽、改造城市道路293条，主城区基本消除了摸黑路，非物业住宅小区得到全面整治，城市管理长效机制逐渐形成，污水、垃圾无害化处理厂等一批重点城建项目建成投入使用。新建、改造县乡客运站38个，完成村通油路2097.6公里，3.8万户农民用上清洁能源，新农村建设收到明显成效。

　　——各项社会事业协调发展。安排国家、省、市科技项目595项，高新技术产品产值年均增长29.4%。中国北方(锦州)农展会成为锦州走向全国乃至世界的一张靓丽名片。文化名城建设硕果累累，4个项目入选国家级非物质文化遗产名录，京剧《酒魂》等一批文化精品荣获国家级大奖，古塔庙会、笔架山海会、国际民间文化节等成为城市品牌。圆满承办了省十运会。广电改革与发展成效显著，节目质量、技术水平、产业实力大幅提升。义务教育均衡发展，凌河区进入省首批“双高普九”地区。职业教育规模不断扩大，办学水平快速提高。城市社区医疗服务体系和城乡突发公共卫生事件应急体系不断完善，成功完成了非典和高致病性禽流感疫情的防控工作。全市人口出生率和自然增长率分别比五年前下降1.24和0.34个千分点。全社会民主法制意识日益增强，信访、社会治安、应急管理、安全生产等工作进一步加强。

　　——人民生活质量明显提高。累计完成棚户区开发改造面积70余万平方米，3.2万人居住条件得到彻底改善。21.8万人实现就业再就业，城镇登记失业率控制在4.8%以内。保障体系趋于完善，社会保险覆盖面进一步扩大，城镇职工参加基本养老、医疗、失业、工伤、生育保险人数分别达到34.3、54.1、39.6、23.7、13.6万人。养老金标准、最低工资标准、城乡低保水平、优抚标准等均有大幅提升。城镇居民人均可支配收入年均增长12.9%，农民人均纯收入年均增长12.3%，城市和农村人均居住面积分别达到24和27.6平方米。

　　各位代表，过去的五年，锦州发生了令人鼓舞的巨大变化，这是省委、省政府和市委正确领导的结果，是全市人民团结奋斗、开拓进取的结果，也得益于历届市委、市政府打下的良好工作基础。在此，我代表市政府，向在各个领域和岗位上辛勤劳动、做出贡献的全市人民表示崇高的敬意！向给予政府工作大力支持的人大代表、政协委员、各民主党派、工商联和各人民团体，向关心和支持政府工作的离退休老同志，向中省直各部门、各单位，向驻锦部队和武警官兵，向所有关心支持锦州发展的海内外朋友，表示衷心的感谢！

　　各位代表，2007年是实现锦州“三年大变样”奋斗目标的决战之年，也是加速辽西沿海经济区中心城市建设的关键一年。一年来，在市委的正确领导下，全市广大干部群众戮力同心、埋头苦干，圆满完成了各项工作任务，确保了“三年大变样”目标的顺利实现。

　　初步核算，全市生产总值实现551.9亿元，增长15.1%。全社会固定资产投资完成167.6亿元，增长37.7%。万元GDP能耗和主要污染物排放量均下降5%。地方财政一般预算收入完成25.9亿元，增长25.9%。实际利用外资1.5亿美元，增长56.9%；实际利用域外资金55亿元，增长22.4%。出口创汇9亿美元，增长37.3%。城镇居民人均可支配收入12010元，增长20.3%；农民人均纯收入5048元，增长21.2%。

　　一年来，我们主要抓了以下十个方面工作：

　　(一)实施沿海发展战略，锦州湾开发势头强劲

　　深入实施省“五点一线”沿海经济带建设战略，“一港、一区、一带、一面”建设取得实质性进展。锦州港总体规划(修编)得到省政府批复，进港主航道浚深工程进展顺利，205十万吨级散杂货泊位竣工运营，西部港区开发一期工程开工建设。港口年吞吐量达到3500万吨，集装箱装卸45万标箱，分别增长11%和45%。开发区建设步伐加快。西海工业区土地整理、道路建设、配套电网和输水管线等工程全部完成，项目承载能力进一步增强，1000吨光纤及半导体用石英玻璃等27个项目开工建设。白沙湾行政生活区基础设施建设快速推进，富海街、玉山路等主要街路竣工通车，一批房地产项目相继开工。凌海沿海经济带规划日趋完善，风力发电等一批项目顺利推进。“港区带”牵动辐射作用不断增强，形成了与内陆县(市)区优势互补、统筹联动的大发展格局。

　　(二)打造新型产业基地，项目建设取得新突破

　　规模以上工业总产值实现806.1亿元，增长25.8%；增加值实现165.9亿元，增长20%；工业固定资产投资实现81.5亿元，增长55.6%。

　　项目建设扎实推进。全市共有373个规模以上项目开工建设，其中3000万元以上132个，亿元以上25个。锦州钛业3万吨钛白粉扩能改造等168个项目竣工投产，锦州石化130万吨加氢裂化等205个项目正在抓紧建设。锦州石化10万吨异丙醇扩建和华润电力两台100万千瓦发电机组等17个项目已着手前期工作，锦赤铁路等一批在谈项目取得实质性进展。

　　基地建设顺利实施。光伏产业列入国家火炬计划特色产业基地，占地1500亩的光伏产业园破土动工，华昌光伏100MW单晶硅太阳能电池等一批项目开工建设，“一园三基地”建设取得重大进展。汽车零部件产业基地建设稳步推进，汉拿电机扩产改造等一批项目竣工达产，占地1000亩的汽车零部件产业园二期工程开工建设，10余家汽车零部件企业有望在年内入驻园区。汤河子工业区建设成效明显，中信铁合金、宝钛华神等一批企业后劲不断增强。

　　县域工业快速发展。西洋特肥、五峰米业等一批企业进一步壮大，黑山国能生物发电、北镇恒亚内燃机配件、凌海金华冶炼扩建1万吨工业硅、义县义兴铁塔等一批重点项目开工建设。

　　国企改革成效显著。铁合金、金纸股权成功出售，华光、重型、矿山等企业资产转让顺利完成，合成纤维厂、女纸等9户企业被列入国家政策性破产企业行列。充分利用国家“减债脱困工程”政策，成功回购了13家国有流通企业资产，化解债务4.8亿元。

　　(三)推动农业全面升级，新农村建设成果喜人

　　农业综合生产能力显著增强，专业化、集约化、标准化、产业化水平进一步提高。预计农业总产值达到200亿元，增长9%。粮食总产量达到200.5万吨。

　　农业全面实现提档升级。农业结构进一步优化，蔬菜、花生、杂粮、水果等优质优势农产品面积扩大到320万亩，海参养殖面积达到4万亩，畜牧业、渔业产值占农业总产值比重分别达到50.5%和11%。增长方式发生实质性改变，棚室总面积达到100万亩，居全省第一位，标准化畜牧小区达到508个，现代农业科技园区达到101个，“三品”认证总量达到248个。经营模式不断创新，规模以上龙头企业达到173户，订单农业面积发展到220万亩，粮食物流总量超过1600万吨，农民专业合作经济组织达到235个。大力开展“阳光工程”培训，转移农村劳动力5.9万人。

　　农村生产生活条件明显改善。治理水土流失面积12万亩，土地开发整理9305亩，基本农田保护率达到87.7%，耕地面积连续9年正增长。农业综合机械化水平达到59%。气象服务能力明显增强，增加水资源2.5亿立方米。建设“绿化新村”128个，新增绿化面积46万亩，森林覆盖率达到24.2%。加大水源地保护力度，汤河子生活垃圾填埋场和防海水入侵主体工程顺利竣工。锦州中心渔港服务功能日趋完善。83%的行政村开通了有线电视。新农村建设“三个一工程”稳步推进，100个示范村建设取得新成果。

　　(四)加强规划建设管理，城市面貌焕然一新

　　城市南扩成效显著。东湖森林公园一期、小凌河第三道橡胶坝、云飞南街女儿河桥等工程即将竣工。松山新区公用设施和9纵5横道路网基本形成。滨海公路开发区段6.4公里竣工通车，7.9公里　　老城区改造和重点工程建设顺利推进。进一步完善城市发展布局，完成了城市总体规划调整、松山新区概念性规划、小凌河和女儿河之间控制性详细规划。新建、大修、拓宽南京路等主次干道21条，汉口街公铁立交桥、锦州公路客运中心站等一批工程顺利竣工。辽宁医学院图书馆工程荣获国家“鲁班奖”。

　　城市管理水平不断提高。64条市管街路全面实行网格化管理，实现了与“门前三包”工作的有效对接。环卫专业队伍长效管理考评机制逐步完善，环卫保洁工作不断向社区延伸。成功迁移南京路旧物市场，店外烧烤、马路市场等城市顽疾得到有效治理。加强环境保护，城市85%以上天数空气质量达到国家二级标准。

　　(五)加快商贸中心建设，区域辐射功能进一步增强

　　商贸中心建设步伐加快，社会消费品零售总额实现185.2亿元，增长17.4%。现代服务业日益繁荣。中兴恒和物流落户锦州，恒大、渤海、华联物流运营良好，恒大物流被评为“中国物流百强企业”。新华广场开业运营，会展中心等项目进展顺利，各类专卖店连锁配送项目发展迅速。农村消费环境明显改善。以“万村千乡市场工程”和“双百市场工程”为重点的现代农村市场体系建设顺利推进，新建、改造、扩建农家店701家，北镇窟窿台和凌海唐家蔬菜批发市场升级改造工作全面完成。新农村网上商品购销对接活动卓有成效。质量与品牌战略取得实效，“五峰牌”大米被评为中国名牌产品。旅游项目开发和精品景区建设力度不断加大，旅游总收入实现56.4亿元，增长44%。居民消费水平明显提高，住房、汽车、休闲度假、旅游观光等渐成新的消费热点。

　　(六)重视财税金融工作，保障服务水平明显提升

　　加强财政对县域经济扶持力度，开展县(市)财源建设综合绩效评价，县域财政实力进一步增强。充分利用财政贴息和税收政策，积极争取上级资金，有效利用国外贷款，大力扶持税源型企业发展，保证了重点项目建设顺利进行。加大重点税源企业、零散税收和非税收入征管力度，严厉打击偷逃骗税等违法行为，税费征管秩序进一步规范。深化国库集中支付制度改革，扩大政府采购规模和范围，财政改革迈出新步伐。调整支出结构，压缩一般性支出，确保了国家收入分配制度改革的落实，保证了企业改制、惠农政策、社会保障等重点支出需求。

　　金融部门在地方经济发展中的作用日益增强。资本市场建设步伐明显加快，汉拿电机在美国纳斯达克全球市场成功上市，阳光能源、中信铁合金等企业上市工作有效推进。市商业银行荣获中国最具竞争力十大城市商业银行奖。农村信用社改革取得阶段性成果。全市金融机构本外币各项存贷款余额均有大幅增长，保险市场经营主体发展到20家。

　　(七)加大招商引资力度，对外开放成效不断显现

　　不断改善投资环境，积极开展招商活动，优化出口产品结构，转变外贸增长方式，对外开放迈出新步伐。成功承办了第六届辽宁台湾周和辽宁·锦州招商引资项目推介会，面向日韩、港台等国家和地区的经贸洽谈活动卓有成效。省外经贸厅项目推进组和我市驻沈阳招商联络处工作成效明显。全年新批外商投资企业44户，汇源果汁、惠发天合等一批项目落户锦州，200万吨水泥熟料加工等一批在谈项目进展顺利。促进现有企业增资扩能，增资项目缴资占全市利用外资的31%。加大奖励、扶持和服务力度，出口创汇平稳增长。继续推行“绿色通道”、企业登记注册“并联审批制”和全程办事代理制，进一步缩短了项目审批时限。县(市)区公共服务中心网络化管理进程不断加快。口岸实现全天候通关，进出口提发货时间缩短到20小时以内。

　　(八)加快文化名城建设，各项社会事业协调发展

　　科技、人才工作创新发展。争取到国家和省立项62项、科技资金2063万元。高新技术产品增加值增长30%。实施国家引智项目18个、地方引智项目40个。教育工作扎实推进。完成了锦州中学与十七中、二职专与三职专合并调整和市卫生学校校舍置换工作，新建农村九年一贯制学校9所，全市中小学实现现代远程教育“校校通”。古塔“双高普九”、义县“巩固普九”和北镇、凌海、开发区“提高普九”提前完成。成功举办了笔架山海会、古玩文化节、辽宁“五点一线”城市电视晚会等大型节庆活动，顺利完成省第七届艺术节锦州主会场承办任务。群众性文艺组织空前活跃，社区周末文化广场等群众文化活动丰富多彩。首届“锦州文化社会办”项目推介会获得圆满成功。北镇荣获“全国文物工作先进县”称号，锦州杂技团节目夺得国际杂技滑稽大赛金奖。广播电视对上报道、获奖总量、经济效益位居全省前列，感动锦州十大电视新闻人物、建设滨海新锦州十大功勋团队电视评选等大型活动受到各界好评。圆满承办了“直通萨格勒布”世乒赛选拔赛、中超辽宁男足主场等26场全国性体育赛事。农村文体广场建设进一步加强。双拥工作成效显著，实现了全国双拥模范城“两连冠”。基层民主建设稳步推进，圆满完成了第九届村委会换届选举工作。人口出生率和自然增长率分别降到5.8‰和1.81‰。残疾人事业长足发展。统计、档案、人防、地震、民族宗教、外事侨务等工作取得新的成绩。

　　(九)实施十项民生工程，和谐锦州建设成效显著

　　全力实施了以平抑物价、农村低保提标扩面、棚户区改造、城乡饮水安全、惠民医疗服务等为主要内容的十项民生工程。猪肉等副食品价格及物价总水平涨幅控制在全省平均水平以下。农村低保标准提高到每人每年1000元，保障面扩大到4%。城镇实名制就业安置10.9万人，确保了“零就业家庭”动态为零，困难群体稳定就业率达到70%。严格市场准入制度，食品、药品监督检查覆盖面均达95%以上。兰花里、兴业里等棚户区改造拆迁工作进入实施阶段。加大城市供水设施改造和农村水源工程建设力度，解决了22万人饮水困难问题。大力开展扶贫开发工程，稳定脱贫人口达到2万人。低保户和改制企业遗留供暖问题得到有效解决。城市社区卫生服务体系和惠民医疗服务体系进一步健全，1所惠民医院、19个社区卫生服务中心、108个社区卫生服务站建成投入使用。农村卫生防保体系不断完善，新型农村合作医疗参合率达到90.8%。归集住房公积金4.8亿元，为完善住房保障体系提供了充足资金储备。公安系统“三基”工程建设全国领先，文化育警工作经验在全国推广。数字化、现代化社会治安防控体系基本建立，司法科研项目成果显著。“平安锦州”建设取得实效，跻身全国50座最稳定城市行列。全面实施“五五”普法规划，广泛开展法制宣传教育活动。制定了突发环境事件、海上搜救等专项应急预案，应急预案体系进一步完善。继续开展领导包案和信访接待日活动，全市群众来信来访件(人)次下降6%。全方位开展安全生产隐患排查治理专项行动，安全生产态势平稳。

　　(十)加强机关作风建设，行政能力不断提高

　　自觉接受人大法律监督、工作监督和政协民主监督，坚持政府与人大代表、政协委员联系制度。全年办理市人大议案1件、人大代表建议和政协提案449件，办复率、满意率分别达到100%和98.4%。全年共受理行政效能投诉189起，办结率达到100%。切实加大监察、审计工作力度，监督水平不断提高。政务公开工作扎实推进，有效促进了阳光政府建设。深入贯彻《公务员法》，加强廉洁自律教育，狠抓机关作风建设，工作实践中涌现出一大批严谨认真、兢兢业业、埋头苦干、积极进取的先进典型。广大干部群众中迸发出的脚踏实地负重奋进的干劲、百折不回锲而不舍的韧劲和不达目的誓不罢休的拼劲，为全市经济社会又好又快发展提供了坚强作风保障。

　　回顾一年来的工作，我们也清醒地认识到，全市经济社会发展还存在一些困难和问题。主要表现在：一是一些主要经济社会发展指标还不是很高，对周边城市和腹地的辐射牵动作用还不够明显，与辽西沿海经济区中心城市的目标要求还有较大差距；二是经济发展方式还比较粗放，科技创新能力不强，科技成果产业化水平较低，国内知名品牌产品稀少；三是县域经济发展还不平衡，有的县(市)落后于全省平均水平，农民收入有待进一步提高，农村整体经济实力亟需快速增强；四是各级财政状况还没有实现根本性好转，财政保障能力有限，工资、社保等刚性支出与可支配财力之间的矛盾仍较突出；五是政府自身建设尚需不断加强，一些干部的思想观念、素质能力、工作作风还不能完全适应锦州湾整体开发开放的需要，软环境建设还存在一些不容忽视的问题，政府依法行政能力也需要进一步提高，等等。这些困难和问题，都需要我们在今后的工作中切实加以解决。

　　二、今后五年工作展望和2008年工作安排

　　各位代表，今后五年是锦州建设辽西沿海经济区中心城市的关键时期。我们一定要全面贯彻党的十七大精神，高举中国特色社会主义伟大旗帜，以邓小平理论和“三个代表”重要思想为指导，深入贯彻落实科学发展观，紧紧围绕建设辽西沿海经济区中心城市的目标，继续实施工业立市、以港兴市和开放牵动三大战略，积极推进改革开放和自主创新，加快经济结构调整和发展方式转变，加强节能减排和环境保护，着力解决民生问题，促进社会事业全面繁荣，实现全市经济社会又好又快发展。

　　今后五年，我们要坚持“两步走”的发展战略，即：经过三年努力，到2010年，全市地区生产总值实现800亿元，提前达到人均生产总值3000美元的小康社会首要指标；再经过两年的奋斗，到2012年，真正把我市建设成为地区生产总值超千亿元的名副其实的辽西沿海经济区中心城市，提前实现“十年大振兴”目标，努力使锦州人民生活得更加美好。

　　按照上述要求，具体工作中，我们要坚持做到“四个牢牢把握”，努力实现“六个提升”。

　　——牢牢把握科学发展这一主题。顺应形势、把握规律，转变发展方式、破解发展难题，促进速度和结构质量效益的统一、经济发展与资源环境的协调，努力实现全市经济社会又好又快地发展。

　　——牢牢把握创新发展这一关键。充分利用我市科技、人才优势，围绕重点领域、关键技术合力攻关，不断增强自主创新能力，加快创新型城市建设步伐，努力使创新发展成为锦州振兴的首要推动力量。

　　——牢牢把握率先发展这一使命。紧紧抓住省“五点一线”沿海经济带建设战略机遇，进一步增强率先发展的使命感和紧迫感，自我加压，奋力争先，努力提高锦州经济社会发展各项指标在辽西沿海经济区中的首位度。

　　——牢牢把握和谐发展这一根本。正确处理改革发展稳定的关系，更加注重社会建设，着力保障和改善民生，扩大公共服务，维护公平正义，加速推进和谐锦州建设进程，努力使改革发展成果更好更多地惠及全市人民。

　　一是实现产业层次的提升。坚持经济结构调整，不断优化三次产业结构，致力构建“现代高效生态农业为基础，新型特色工业为主体，现代服务业为拉动”的产业发展格局。到2012年，全社会固定资产投资年均增长23%以上，三次产业结构由2007年的20.5∶39.9∶39.6调整为13∶45∶42，努力形成经济运行加快、经济增长协调、经济结构优化、经济社会和谐发展的良好态势。

　　二是实现开放程度的提升。坚持软、硬环境建设，扩大开放领域，提高利用外资质量，优化出口商品结构，实施“走出去”战略，加速推进锦州湾整体开发，致力构建“全方位、高水平、宽领域”的大开放格局。到2012年，全市对外开放度明显提高，实际利用外资、出口创汇年均分别增长20%和10%，实际利用外资继续保持高于辽西其它各市总量的领先地位。

　　三是实现城市品位的提升。坚持科学城市定位，高标准实施新一轮老城区改造，加速推进城市南扩，加大南站地区和白沙湾行政生活区开发建设力度，致力建设“思想观念更新、城市布局更优、文明程度更高”的滨海新锦州。到2012年，努力把锦州建设成为拥有百万人口、百平方公里城区和亿吨大港的山水相依、城海相连的现代化港口城市。

　　四是实现财政实力的提升。坚持税源建设，强化依法征税，深化财税改革，严格财税管理，致力形成“财源雄厚、配置高效、运行规范”的财政运行体系。到2012年，地方财政一般预算收入突破50亿元，自有可支配财力增强，政府保证政权运转、社会稳定和支持事业发展的能力大幅提升，全市财政特别是县域财政困难状况有效缓解，“吃饭财政”的被动局面基本改变。

　　五是实现生活质量的提升。坚持以人为本，着力解决事关群众切身利益的现实问题，有效提高人民生活水平，致力建设“环境优美宜居、百姓安居乐业、社会稳定和谐”的小康社会。到2012年，群众生活质量实现大幅提升，城镇登记失业率控制在4.5%以内，城市居民人均可支配收入和农民人均纯收入超过全省平均水平，社会保障体系更加完善，城乡环境更加优美，文化氛围更加浓厚，平安锦州建设更加富有成效。

　　六是实现行政效能的提升。坚持执政为民，转变政府职能，强化依法行政，推进效能建设，致力打造“开拓创新、务实高效、清正廉明”的服务型政府。通过五年的努力，着力造就一支雷厉风行、令行禁止的“想干事、会干事、干成事”的公务员队伍，切实增强政府的执行力和公信力。

　　各位代表，2008年是新一届政府任期的开局之年，做好今年各项工作，对于加速辽西沿海经济区中心城市建设，提前实现“十年大振兴”奋斗目标，具有十分重要的意义。

　　2008年经济社会发展的主要预期目标：地区生产总值增长15%以上，一、二、三产业结构调整到18.3∶41.2∶40.5，万元GDP能耗和主要污染物排放量均降低5%左右，全社会固定资产投资增长23%，地方财政一般预算收入增长15%，实际利用外资和域外资金分别增长20%和15%，出口创汇增长10%，社会消费品零售总额增长12%，物价总水平涨幅控制在全省平均水平，城市居民人均可支配收入和农民人均纯收入分别增长11%和12%，城镇登记失业率控制在5%以内。

　　围绕上述目标要求，重点做好以下十个方面工作：

　　(一)坚持沿海发展战略，加快锦州湾整体开发

　　加快港口开发建设，增强锦州港牵动能力。推进港口泊位及航道浚深等配套设施建设，加快西部港区开发进程。完成锦州港西、南防波堤及围堰一期工程和三港池建设，确保206散杂货泊位竣工试运营，完成209、210集装箱泊位建设前期工作，规划建设3个10万吨级和5个5万吨级煤炭专业化泊位。挖掘本地货源，开拓腹地市场，扩大国铁货运直进直出运量，确保港口吞吐量增长20%以上，争取实现设计能力。加强中蒙经贸合作，推进锦州港作为蒙古国出海口建设进程。

　　围绕“三园一带一中心”建设，加快开发区提档升级。继续完善西海国际工业园、白马综合工业园和白沙湾行政生活区配套工程建设，重点推进华润电力两台100万千瓦发电机组等一批项目开工建设，切实做好锦赤铁路等项目前期准备工作，力争国家石油战略储备基地等一批项目取得实质性进展。着力推进开发区晋升国家级开发区、保税物流中心和区港联动试点申报工作。

　　加大项目引进力度，推动凌海沿海经济带建设。加快滨海公路等基础设施建设，广泛吸引投资者，大力发展农产品深加工、金属冶炼、海水养殖、风力发电、滨海旅游和商贸物流业，建设沿海特色产业集群。

　　统筹联动共享资源，提升“港区带”辐射能力。探索建立“港区带”与腹地间多层次、多形式合作的新机制，加快形成各具特色、优势互补的区域经济发展新格局，全面带动黑山、北镇、义县等县(市)区整体融入锦州湾开发开放。

　　(二)提升项目建设水平，推动工业持续发展

　　注重质量增加效益，加快项目建设。支持锦州石化、中信铁合金、元成玉米等重点企业做强做大。全力促成宏丰6000万米印染布等59个重点项目竣工投产，10万吨异丙醇等146个项目开工建设。力争新增规模以上企业120户。确保规模以上工业增加值增长17%，工业固定资产投资增长20%。

　　扩大规模优化结构，培育产业集群。围绕“324”产业格局，重点建设石化、光伏、汽车零部件、精细化工等10大新型产业基地和30个工业园区，推进华昌光伏100MW太阳能电池、金华冶炼1000吨多晶硅、新世纪石英玻璃1000吨低成本多晶硅、日鑫硅1000吨单晶硅、天合化工1000吨氟碳醇等项目尽快达产。

　　因地制宜突出特色，壮大县域工业。加快黑大、沟帮子、双羊、七里河、汤河子等工业园区建设，进一步增强园区承载力和吸引力。培育支柱产业，做强骨干企业，打造名牌产品，形成3-5个竞争力强的主导产业，新增5-10个税收超千万元的骨干企业。

　　坚持标准落实责任，实行节能减排。建立健全节能减排指标、监测和考核体系，落实专项资金和目标责任制，坚决控制新建高耗能、高污染项目。依法开展违法排污专项整治，严厉打击环境违法行为。按照建设资源节约型、环境友好型社会的总体要求，切实转变观念、形成共识，努力在全社会形成节约集约资源和保护环境的良好氛围。

　　(三)大力发展现代农业，扎实推进新农村建设

　　提高科技应用水平，加快现代农业发展。新增水浇地10万亩、棚室5万亩，建设高标准畜牧小区100个。进一步提高重大动物疫病防控能力，确保无重大动物疫情发生。推广农业新品种、新技术100项，优良品种覆盖率达到98%以上。新增“三品”认证30个。大力发展农产品加工业，促进龙头企业集群化发展，新增规模以上龙头企业20户，发展订单农业240万亩。培育现代农业经营主体，发展新型农民专业合作社60个。粮食物流总量实现1650万吨。农业综合机械化水平提高到60%。继续实施增雨工程，增强气象防灾减灾能力。深入开展“阳光工程”培训，新增转移农村劳动力5万人。

　　加强基础设施建设，进一步改善农村生产生活条件。加大耕地保护力度，节约集约利用土地，严厉打击土地违法行为。改善治涝面积10万亩，治理水土流失面积12万亩。人工造林25万亩，封山育林10万亩，建设“绿化新村”100个。加大水源地保护力度，继续推进农村饮水安全工程。完成村通油路345.7公里，实现所有行政村通油路。力争实现2.5万农村贫困人口稳定脱贫。加快新农村电气化工程建设。增加清洁能源用户1万户。实施“村企共建工程”，开展“千村整洁行动”，推进100个新农村建设示范村达标升级。

　　(四)全面加快城市建设，构建优美宜居环境

　　继续推进城市南扩。开工建设云飞南街小凌河桥以及2座跨小凌河步行桥，按时完成二热三期、松山新区公交场站、自来水和燃气管网铺设等工程。启动实施连通锦葫两港的锦州湾大桥工程，完成开发区段滨海路建设。

　　切实加快老城区改造。新建、拓宽、改造凌西大街、云飞南街、宜昌路等15条主要街路。做好新一轮老城区改造规划工作。启动实施锦州石化职工住宅项目，推进兰花里、兴业里、万年里、太和里等棚户区改造进程。全面接收和改造二次供水系统70座。实施中水回用工程。开工建设5.2公里锦汤路。

　　不断提高城市管理水平。进一步理顺城市管理体制，建立健全城市管理长效机制，发挥城市管理监督员作用，加快“门前三包”和网格化管理向街道、社区延伸。扩大拆墙透绿成果，完善亮化设施。深入开展创建优美住宅区活动，进一步改善人居生活环境。

　　(五)发展壮大现代服务业，培育新的经济增长点

　　注重消费对经济增长的拉动作用，加快服务业发展。加速会展中心、五星级酒店、锦州港粮食物流二期、恒大物流及保税中心等项目建设，推进物流、信息、配送、会展、中介、理财等新型服务业蓬勃发展。加大传统服务业升级改造力度，积极引进国外知名企业入驻锦州，努力增加流通服务业经济总量。继续实施“万村千乡市场工程”和“双百市场工程”，构建新型农村流通网络和商业服务体系。加强县乡中心店和商品配送中心建设，增强配送能力，提高商品质量。重点抓好各类专业批发市场建设，不断增强大型专业市场集散功能。整合旅游资源，加快项目开发，打造精品线路，强化宣传促销，发展壮大红色旅游和乡村旅游，促进全市旅游业快速发展。

　　(六)强化财税金融工作，提高财政运行质量

　　稳固税源基础，提升财税增收能力。大力培植财源，支持产业结构调整，建立互补性税源。继续实施县乡财源建设工程，做大财政收入规模，规范财税法律法规秩序，增加县乡可支配财力，推进县域财政位次前移。依法组织财政收入，加大对重点地区、企业、税种监控力度，严厉打击偷逃骗税等违法行为，避免税收流失，做到应收尽收。

　　强化预算管理，提升财政保障能力。推进财税改革，逐步完善公共财政体系，整合政府财力资源。继续加大公共服务领域投入，着力改善民生，推进基本公共服务均等化。狠抓增收节支，优化财政支出结构，保证财政平稳运行。加强对收入征管和财政运行的审计监督，提高税费征缴入库率和财政资金使用效益。

　　完善经营体系，提升金融服务能力。搭建银企对接平台，搞好中小企业融资服务，着力解决制约民营经济发展的资金“瓶颈”问题。增强资本市场融资功能，加快阳光能源、中信铁合金、商业银行等企业上市进程。加强金融生态环境建设，打造诚信锦州，吸引外资银行入驻我市。推进保险行业诚信服务和保险市场健康发展。

　　(七)深化改革扩大开放，开创对外开放新局面

　　加大攻坚力度，推进各项改革。加速推进合成纤维厂、女纸等9户企业政策性破产工作，切实搞好辽晶、包装机械厂等企业资产重组和优化，妥善做好金纸、叉车等改制企业的后续工作。加大国有资产监管力度，严防国有资产流失。积极做好股权管理和债权追索工作，最大限度地盘活存量资产。稳妥推进公用事业改革，加大交通、商业、粮食、文化等行业改革推进力度。

　　拓宽招商渠道，提高引资质量。充分发挥省外经贸厅项目推进组、我市驻沈阳招商联络处及相关部门作用，巩固发展好同日韩、港台等重点国家和地区的合资合作，扎实开展好珠三角、长三角以及沈阳、大连等地的招商活动。实际利用外资力争突破1.8亿美元。筹备建设锦州湾口岸综合查验大楼，推进口岸查验“一站式”办公和电子化网上办公进程，进一步改善通关环境。

　　优化出口结构，扩大出口规模。用好用足政策，加大对锦州石化、沈宏、东方集装箱等出口龙头企业的扶持力度。重点抓好机电、光伏、农产品深加工等高技术含量、高附加值产品的出口，努力扩大技术出口和劳务输出。

　　(八)加快社会事业发展，提升公共服务能力

　　加强科技工作和人才队伍建设。提高自主创新能力，发展高新技术产业，促进产业结构调整和传统产业升级。加大科技投入，推进重大科技项目攻关。开展创建技术创新型企业活动，加快建立技术创新体系，加速成果转化和专利技术商品化、产业化进程。拓展培养、吸引、使用人才领域，加强高级研发人才、复合型人才和技能型人才队伍建设。

　　加快教育和文化体育事业发展。进一步推进义务教育均衡发展，新建农村九年一贯制学校8所，力促凌海“双高普九”和黑山、太和“提高普九”通过省评估验收。重视学前教育，大力发展职业教育。继续办好笔架山海会等大型节庆活动和社区周末文化广场等群众性文化活动。加快文化产业发展，繁荣公益性文化事业。积极推进广播电视“村村通”和广播电视数字化工程。加强体育设施建设，广泛开展“迎奥运”活动，办好各类体育赛事，大力发展群众性体育运动，不断提高全民身体素质。深入开展群众性精神文明创建活动，培育文明风尚，努力形成人人促进和谐的良好局面。

　　抓好医疗卫生等工作。不断完善惠民医疗制度，切实提高城乡医疗服务水平。新型农村合作医疗常住农业人口参合率和基金使用率分别达到100%和95%。继续加强社区卫生服务机构建设与管理。全面落实农村计划生育奖励扶助政策，人口出生率和自然增长率分别控制在8.99‰和3.85‰以内。认真开展第二次全国经济普查。进一步做好档案、人防、地震等工作，积极发挥电业、通讯、烟草等中省直单位作用。

　　(九)维护群众切身利益，推进和谐锦州建设

　　广开就业渠道，提高保障水平。实现实名制就业5.8万人，其中安置大龄就业困难人员5000人，确保零就业家庭每户至少有一人实现稳定就业，棚户区等困难群体稳定就业率达到70%以上。积极做好城镇其他失业人员、进城务工人员和新生劳动力特别是大中专毕业生就业创业工作。实施普惠制培训3万人，劳务输出3万人。加快实施困难企业退休人员医保、被征地农民保障、农村医疗和养老保险等惠民政策。全面落实“4050”困难人员社会保险补贴政策。启动城镇居民医疗保险。全面贯彻《就业促进法》和《劳动合同法》。加大社会救助力度，切实解决特殊困难群体就医、上学、取暖等问题，认真做好城镇低收入家庭廉租住房建设工作。积极推进慈善和残疾人事业发展。

　　维护社会稳定，创造和谐环境。巩固提高公安系统“三基”工程建设成果，加强社会治安、道路交通和消防安全管理，依法打击处理各类违法犯罪，有效处置群体事件和突发事件，全力打造和谐平安锦州。落实安全生产责任目标，全面提高安全生产管理水平，坚决遏制重特大安全生产事故发生。进一步健全矛盾纠纷排查化解机制，确保群众合法利益诉求和信访群众的实际困难得到有效解决。深入开展普法教育，做好法律服务和法律援助工作。巩固双拥工作成果，落实各项优抚安置政策。发挥民族宗教在经济发展与和谐社会建设中的积极作用。

　　(十)加强政府自身建设，提高依法行政能力

　　按照“求真务实，雷厉风行，令行禁止，严细成风”要求，进一步加强机关作风建设。严格执行人大及其常委会的决议、决定，切实完善工作报告制度，自觉接受人大依法监督、政协民主监督和人民群众的社会监督，努力做好人大代表议案、建议和政协提案办理工作。积极支持工会、共青团、妇联等人民团体的工作。认真落实党风廉政建设责任制，严格执行廉洁自律各项规定。加大行政监察、审计监督力度，深入开展政风行风评议，规范各级政府和公务员的行政行为。建立健全各级政府部门协调配合机制，不断增强解读运用政策能力，切实解决政出多门、推诿扯皮等问题。加快电子政务和政府网站建设，扎实推进政务公开和村务公开，适时开展第四次行政审批制度改革。创新服务体制，改进服务方式，完善群众诉求机制，不断拓宽群众与政府的沟通渠道，努力为群众提供更好更多的公共服务，真正实现好、维护好、发展好人民群众的根本利益。

　　各位代表，新的征程赋予我们新的使命，新的起点给予我们新的希望。让我们在市委的坚强领导下，紧紧团结和依靠全市人民，奋力拼搏，锐意创新，为创造锦州人民更加美好的生活而努力奋斗！
